# Use and future of wiki systems in veterinary education? – A survey of lecturers in German-speaking countries

**DOI:** 10.3205/zma000996

**Published:** 2015-11-16

**Authors:** Darius Kolski, Wolfgang Heuwieser, Sebastian Arlt

**Affiliations:** 1 FU Berlin, Tierklinik für Fortpflanzung, Berlin, Deutschland

**Keywords:** E-learning, Education, Wiki, Lecturers, Veterinary medicine

## Abstract

**Objectives: **Wiki systems are becoming increasingly important in university teaching. Not much is known about the opinion of lecturers of veterinary medicine regarding the active participation of students in teaching, their opinion on wiki systems and their motivation to use them in courses and to improve the quality of information. The objective of the present study was to evaluate how lecturers of veterinary medicine estimate learning management systems and the production of text or material by students in courses, if they rate wiki systems as an appropriate tool for teaching, if they would use wiki systems for their courses and if they are willing to improve the quality of information.

**Methods: **The data collection was carried out as an online survey using a five-point Likert scale. Lecturers of veterinary medicine in Germany, Austria and Switzerland were contacted (n=approx. 1700) out of which 139 completed (8.2%) the survey.

**Results: **Most lecturers use LMS and consider it to be suitable for providing course material. Half of all respondents indicated that they believe that students achieve greater learning success by developing their own learning material. In courses 23.0% of their students develop own materials. The majority of lecturers considered wiki systems as an appropriate and complementary tool for teaching (53.6%). A collection of wiki articles is seen as useful (56.6%), particularly when experts review the contents. One third of the lecturers would use wiki systems for the creation of material by students, but 82.5% have not yet used them in teaching. One third is willing to participate in the review of articles with regard to their quality.

**Conclusion: **The results show that many lecturers are willing to use veterinary wiki systems and that they regard them useful for teaching. According to the opinion of the majority of lecturers, the creation of material by students can lead to greater learning success and wiki systems are suitable for this purpose. We are about to develop strategies to support the implementation of wiki systems into veterinary education and a peer review system supported by lecturers. In a further project the actual learning success provided by the active use of wiki-systems by students will be evaluated.

## Zusammenfassung

**Zielsetzung: **Wiki-Systeme gewinnen in der universitären Lehre zunehmend an Bedeutung. Jedoch sind die Ansichten von Dozierenden der Veterinärmedizin in Bezug auf eine aktive Beteiligung der Studierenden in der Lehre, zu Wiki-Systemen und deren Nutzung im Rahmen von Lehrveranstaltungen und zur Qualität der Informationen in Wiki-Systemen wenig bekannt. Das Ziel der vorliegenden Studie war es zu bewerten, wie Dozierende der Veterinärmedizin Lernplattformen (learning management systems, LMS) und die Erstellung von Texten oder anderen Materialien durch Studierende einschätzen. Weiterhin wollten wir erfragen, ob sie Wiki-Systeme für ein geeignetes Instrument für den Unterricht halten, ob sie diese in ihren Kursen nutzen würden und ob sie bereit wären, die Qualität von Informationen in Wiki-Systemen zu verbessern.

**Methodik:** Die Datenerhebung wurde anhand einer Online-Umfrage mit einer Fünf Punkte Likert-Skala durchgeführt. Dozierende der Veterinärmedizin in Deutschland, Österreich und der Schweiz wurden kontaktiert (n=ca. 1700), von denen 139 (8.2%) an der Umfrage teilnahmen.

**Ergebnisse: **Die meisten Dozierenden nutzten LMS und hielten sie für geeignet, um Kursmaterial bereitzustellen. Die Hälfte der Befragten glaubte, dass die Studierenden einen größeren Lernerfolg durch die Entwicklung ihrer eigenen Lernmaterialien erzielen. Insgesamt gaben 23.0% an, dass Studierende eigene Materialien in ihren Kursen erstellen. Die Mehrzahl der Dozierenden betrachtete Wiki-Systeme als ein geeignetes und ergänzendes Instrument für den Unterricht (53.6%). Eine Sammlung von Wiki-Artikeln wurde als nützlich angesehen (56.6%), vor allem, wenn Experten den Inhalt begutachtet haben. Ein Drittel der Dozierenden würde Wiki-Systeme für die Erstellung von Materialien durch Studierende verwenden, aber 82.5% haben sie noch nicht in der Lehre verwendet. Ein Drittel wäre bereit, sich bei der Begutachtung der Artikel im Hinblick auf ihre Qualität zu beteiligen.

**Schlussfolgerung: **Die Ergebnisse zeigen, dass viele Dozierende bereit sind, veterinärmedizinische Wiki-Systeme zu verwenden und dass sie diese für die Lehre als nützlich und geeignet betrachten. Nach der Meinung der Mehrheit der Dozierenden kann die Erstellung von Materialien durch die Studierenden zu einem größeren Lernerfolg führen. Derzeit werden Strategien entwickelt, um die Implementierung von Wiki-Systemen in der tierärztlichen Ausbildung und ein Begutachtungs-System durch Dozierende zu unterstützen. In einem weiteren Projekt soll der tatsächliche Lernerfolg durch die aktive Nutzung von Wiki-Systemen durch Studierende ausgewertet werden.

## 1. Introduction

The use of the Internet in university educational environments has seen a significant increase in recent years [1]. Universities and lecturers are increasingly using for example learning management systems (LMS) [2], [3], [4], with which teachers can make course materials available and communicate with students [5].

Modern educational concepts attempt to give students a more active role. The aim is to set the focus on students and encourage team-oriented, collaborative learning and interaction between them. The roles of teachers and learners are thus partly modified, as students develop materials such as texts, images, and presentations to teach other [5]. In implementing these concepts, Web2.0 technologies such as wiki systems and blogs are becoming increasingly important. A wiki system is a website that contains a collection of linked websites. These websites can be developed and edited by individuals or a group of users working collaboratively [6], [7]. This important feature of a wiki system to edit content by all users gives the opportunity that articles can be corrected, updated or completed. 

The number of wiki-in-education related projects and publications has increased considerably in recent years [8]. Some authors regard wiki systems as a suitable tool for university teaching [8], [9]. Wiki systems allow lecturers to develop interactive activities for their students, and to present various course information such as texts, images, videos, literature sources, external links, project information, and frequently asked questions (FAQs) [10]. Wiki systems support the creation of texts because they are rapidly deployed and easy to use [11]. 

Some authors see a potential of wiki systems to become platforms for large and up-to-date knowledge repositories, because they engage a potentially large group into the knowledge creation process [8], [12]. A challenge in the use of wiki systems in teaching is to ensure the quality of the content developed [13]. On the one hand, the option of editing information is an advantage because errors can be corrected. On the other hand, there is the risk that correct information can be supplemented or replaced by inaccurate or false information. Wiki content is generally not reviewed by experts (e.g., peer review) prior to its publication [12]. The objective of the present study was to evaluate how lecturers of veterinary medicine estimate learning management systems and the production of text or material by students in courses. Furthermore we wanted to know whether they rate wiki systems as an appropriate tool for teaching, if they would use wiki systems for their courses and if they are willing to improve the quality of information. 

## 2. Materials and Methods

In May 2013, emails were sent to the professors and research assistants employed by the veterinary medicine universities in Germany, Austria and Switzerland (Berlin, Giessen, Hannover, Leipzig, Munich, Vienna and Vetsuisse). The email contained a link to the survey and a uniform password for all participants. The option to participate ended on 28 June 2013.

The survey was carried out using an online questionnaire. The software EFS Survey at Quest Back, Köln-Hürth, Germany was used. The password was intended to prevent uninvited users from accessing the questionnaire.

A total of approximately 1700 lecturers were contacted. Furthermore we sent emails via the deans office of the respective university with the question to distribute these questionnaire to research assistants. We do not know how many lecturers were actively contacted through the deans and received the questionnaire. Therefore we estimate that about 1700 lecturers were contacted. Of the contacted lecturers, 139 (8.2%) participated in the survey.

The questionnaire (see attachment ) contained a brief introduction to the study as well as questions regarding age, professional status, working subject and university.

In addition, the participants were asked to agree to or reject specified statements using a five-point Likert scale. The first part of the questionnaire was related to material made available online by lecturers, mainly by LMS. It also contained questions related to the development of content by students in university courses.

The second part of the questionnaire dealt with the use and acceptance of wiki systems in courses. It contained 12 statements about the attitude of lecturers toward wiki systems. The questionnaire also included questions about the quality of information in a wiki system. 

The analysis was performed using the statistical program SPSS^®^ (Statistics IBM® version 20, Armonk, New York, USA). Data were analysed based on frequencies of answers and frequency distributions. Furthermore, lecturer´s answers were analysed according to the response of their particular group (professor or research assistant) using the chi-square test, expected frequencies and standardized residuals for correlations. The significance level was set as a=0.05. The population represented (n) varied depending on the number of lecturers who had answered the respective questions.

For the presentation in this article the results from the statements "I strongly agree" and "I agree" and the results from the statements "I do not agree" and "I strongly disagree" were added together, respectively, to form one affirmative and one negative statement. The statement “neutral” means that the respondent does not support or decline a statement and it is offered to avoid that the lecturers leave the question blank. The statement “undecided” means that she or he is not able to assess a statement or is not willing to do so.

## 3. Results

A total of 139 lecturers completed the survey, of which 96 were research assistants (70.1%) and 41 professors (29.9%). Two people did not specify their professional status. There is a shift with respect to the responses. Almost half of the responses (40.2%) was carried out by the 3 universities from Austria and Switzerland. The Freie Universität Berlin had the highest response rate regarding the German universities. A number of 29.9% of the replies were made by professors who make up only about 10.0% of the population of respondents. 

A large number of lecturers (73.4%) indicated that they regularly make material for their lectures available online. Of these lecturers, 43.1% used LMS. More professors than research assistants stated that they regularly made material for their lectures available online (p<0.01). Professors also used LMS more frequently for this purpose (p< 0.01). 64.0% of the surveyed lecturers considered LMS to be suitable for providing course material. 

In total, 23.0% of lecturers indicated that students develop their own texts or other learning material in the context of their courses. Out of these, 49.3% stated that students did work on their texts not within lecture times. A total of 45.3% were of the opinion that students achieve greater learning success if they develop their own texts and learning material. However, 36.7% of the lecturers stated that they could not evaluate this statement. The majority of the lecturers stated that they perceive it as useful that the material produced by students can be used in subsequent courses (55.0%) and revised by students (45.7%). 46.1% of the participants disagreed with the statement that the study of veterinary medicine allow adequate time for students to independently edit content. 

The majority of lecturers regarded it useful to have a collection of wiki articles for veterinary medicine as a source of information (56.6%). The lecturers considered wiki systems as an appropriate and complementary tool for teaching (53.6%).

Only 31.0% of the veterinary lecturers would use a veterinary wiki system for the creation of material by students. 41.9% were neutral to the statement.

Most participants (82.5%) had not previously used wiki systems for the creation of material by students. 25.8% of lecturers would write or revise articles in a veterinary wiki system and 38.5% would like to participate in the review of articles to improve the quality of articles in veterinary wiki systems. 

A total of 32.8% of the lecturers had concerns regarding the quality of the information in wiki systems. Most participants (88.1%) thought that experts should review the information in a veterinary wiki system prior to its publication. 

One third (69.2%) of lecturers indicated that the linking between wiki articles could lead to a better understanding of interdisciplinary contexts. More research assistants (76%) than professors (43.9%) agreed with this statement (p<0.01). More than half of the lecturers (66.9%) considered the opportunity of editing and updating articles as an advantage, while 28.7% as a disadvantage. A total 52.9% of lecturers favoured a non-public wiki system, 18.1% chose the statement "neutral".

Towards many statements the respondents were "neutral” or “undecided". The statement "neutral" was chosen more often than the statement "undecided". Six of nine neutral statements of the first questionnaires had values between 15.1% and 28.8%, eight of twelve "neutral-/undecided" statements had values between 24.3% and 46.3%. Especially the statements regarding the use a veterinary wiki system for the creation of material by students, if lecturers would write or revise articles in a veterinary wiki system and if they see the opportunity of editing articles as a disadvantage had values around and over 40.0% (see Table 1 (Tab. 1), Table 2 (Tab. 2) and Table 3 (Tab. 3)).

## 4. Discussion

This survey was designed in the context of an increasing use of wiki systems at universities [14] and the related issues regarding the quality of the information. Generally, the survey results show that lecturers support active participation of students in lectures and consider the use of wiki systems as an appropriate tool for teaching. Furthermore, they are willing to improve the quality of information. Nearly half of the lecturers thought that students achieve greater learning success through active participation and that material produced by students should be used in subsequent courses. More than half of the lecturers think it is useful to have a collection of wiki articles for veterinary medicine (56.6%) and one third of the lecturers would use a veterinary wiki system for the creation of material by students. An amount of 38.5% would like to participate in the review of articles to improve the quality of articles. 

Many professional medical wiki knowledge bases are already available. Examples are ganfyd.org, a free medical knowledge base, which any registered medical practitioner can edit [http://www.ganfyd.org/index.php?title=Main_Page] or radiopaedia.org, a growing and free educational radiology resource [http://radiopaedia.org/]. The motivation to use wiki systems was expressed by approximately one third of the lecturers. We speculate that the low motivation of the other respondents is due to the fact that German wiki systems were hardly available or hardly known in the past. Barely half of lecturers of veterinary medicine use LMS to provide material online. According to a survey conducted in Sweden [3], lecturers use LMS predominantly to distribute documents to students and to facilitate their existing teaching practice. LMS at the Freie Universität Berlin offer the possibility to share documents and information but do not offer the option of collaborative creation or the editing of content unlike wiki systems. Wiki systems support the creation of texts during and outside of courses. They can provide an efficient and flexible interface for knowledge creation and student interaction [10]. They enable the lecturers to inspect the results at any time. The most active teaching technique is the in-class activity, as it leads to a better understanding of course materials [15]. If appropriate, wiki systems may be used during the lecture times or to supplement teaching outside of normal lecture times. Furthermore, it may be advantageous to provide adequate time during the course so that the students can develop wiki articles. This suggestion is relevant in the context of the opinion of half of the lecturers that the study of veterinary medicine offers insufficient time periods for self-study. Another argument for the use of wiki systems in veterinary teaching is that the majority of lecturers would use the developed material in subsequent courses and would let the students revise the articles. 

Aspects regarding the quality of the information are an important issue in wiki systems used by lecturers. One third of lecturers have concerns regarding the quality of the information in wiki systems. Since its founding, the online encyclopaedia Wikipedia, is regularly criticized by academics as being tawdry and full of inaccuracies [16]. It is difficult for many visitors to trust the content in Wikipedia because of the high variance in quality of Wikipedia articles [17]. However, concerns regarding the quality of the information in wiki systems have not been supported by other studies. The Wikipedia community takes issues of quality very seriously. Even though anyone can edit articles, the results are carefully discussed and there is an intense, on-going review of articles [18], [19]. Wikipedia is regarded as an accurate and comprehensive source of drug-related information for undergraduate medical education [20]. One approach to improve the quality of information is the identification of articles of high quality by specialized experts as "good" articles [21] or “featured” articles [17]. This identification of articles in a veterinary wiki system could be done through a review process, which is mainly carried out by lecturers. It is encouraging that about one third of the lecturers would participate in a review of articles in a veterinary wiki system. Some lecturers indicated that they are willing to participate in the creation or revision of articles and the technical features are in progress. Almost all the lecturers thought that experts should review the information prior to its publication. Professional wiki systems like Radiopedia developed a board of editors to control the accurateness of the information [http://radiopaedia.org/]. Despite the large amount of high-quality information available on Wikipedia without a permanent appraisal [22], a review process in a veterinary wiki system is advisable. Especially wiki projects, which are to be integrated into teaching, should establish a peer review process, for example because of sensitive information such as the diagnosis or treatment of disease [13]. A proven concept is the graded peer review process in which an article can have four types of status: Incomplete (development of the article is in progress), Published (articles are published without prior examination and can then be annotated, modified, supplemented and corrected by each participant), Peer-reviewed (the evaluation of the article is done by ordinary users ("peers") and students with the help of a review guide on the talk page) and Expert Review (reviewed by experts) [13]. After completion of a successful expert review, the article can be protected from further editing, in order to verify the accuracy of information. Modifications are only possible after a request to the moderator. 

The complexity of information and skills in medicine has increased and led to an increasing specialization within the health professions [23]. The opportunity for interdisciplinary exchange is becoming smaller because of the increasing specialization [24]. Wiki systems offer the possibility of linking content. Thus, information can be found faster and interdisciplinary learning is thereby facilitated. The majority of lecturers (69.2%) also saw an opportunity in the linking of wiki articles to promote a better understanding of interdisciplinary contexts. More research assistants than professors stated that they support this statement (p<0.01). This may be an indication that research assistants in particular regard the interdisciplinary potential of wiki systems as an advantage. Articles with a higher number of links attract a larger number of contributors, and potentially have more experts involved, which may result in a higher quality of articles [25]. Regarding teaching, links may enable a better understanding of the context of the information presented in an article. 

Almost two-thirds of the lecturers see the opportunity of editing articles as an advantage. The opportunity to edit articles on Wikipedia is considered as its most controversial advantage, because all entries are collectively developed by the global community of Wikipedia users [26]. However, this is also the greatest means of updating information. It is important to have the option of updating information, especially for medicine, a science where the amount of information is greatly increasing [27].

However, 28.7% of the lecturers see the opportunity of editing articles as a disadvantage because incorrect information can be added. The appearance of incorrect information may have different reasons, for example vandalism or lack of expertise. Vandalism rarely appears in wiki systems used in education [14]. Almost half of vandal contributions are repaired within one view [19]. In addition, earlier versions of the article are easy to restore in a wiki system. The lack of expertise of student writers could be controlled by a review system. A workflow would have to be implemented in that articles could have a “not reviewed” or “reviewed” status. The use of a veterinary wiki system may be limited to members of the veterinary medicine community as supported by about half of the respondents. Other lecturers clearly see the openness of a wiki system as a benefit. A public domain system would allow animal owners and members of medical or agricultural professions to have access to the information. A survey of students in Switzerland also showed that students favour the openness of a wiki system [14]. A solution can be a semi public wiki system, where the content can only be edited and read by registered users, but selected reviewed content is public. Currently, a German-language wiki system for veterinary medicine is being developed (http://www.vetipedia.org), which will be established as a semi public wiki system. 

This survey cannot be regarded as representative. The proportion of 8.2% respondents of the initially contacted persons is fairly low. Physician surveys are an important tool in health services, but they are often characterized by low response rates [28]. A study of Australian doctors’ use of online social media had a slightly higher response rate of 12.47% [29]. It remains unclear if lecturers who are interested in online teaching and wiki systems were more likely to participate. Nevertheless, 139 lecturers participated in the survey. Most respondents were members of the universities of Vienna, Swiss (Bern and Zürich) and Berlin. The lowest response rates had the universities of Leipzig and Giessen. A reason could be that the proportion of contacted lecturers varied. In relation to the amount of research assistants in German-speaking universities, more professors filled out the questionnaire. It must also be noted that only lecturers in German-speaking veterinary medicine universities were consulted. Another phenomenon is the high rate of neutral or undecided statements, which may have different reasons. Many lecturers in veterinary medicine may have little experience with Web 2.0 technologies and were, therefore, not able to assess the statements. In addition, a study on bias in surveys found that a neutral scale position that is included in a questionnaire increases the number of neutral responses compared to the same survey without a neutral scale position [30]. 

Despite these limitations we consider the results of the present study relevant in order to evaluate the views and motivations of the lecturers, and to develop practical concepts for the application of a veterinary wiki system in teaching.

## 5. Conclusion

This data shows that many lecturers are willing to use veterinary wiki systems and that they regard them as useful systems. One fourth stated that they are also willing to actively participate in article writing and revising. According to the opinion of the majority of lecturers, the creation of material by students can lead to greater learning success and wiki systems are suitable for this purpose. We are about to develop strategies to support the implementation of wiki-systems into veterinary education and a peer review system supported by lecturers. This encompasses also tutorials and scenarios for lecturers and other helping material that aims to address possible constraints of the media skills. In a further project the actual learning success provided by the active use of wiki systems by students will be evaluated. 

## Competing interests

The authors declare that they have no competing interests.

## Supplementary Material

Questionnaire

## Figures and Tables

**Table 1:**
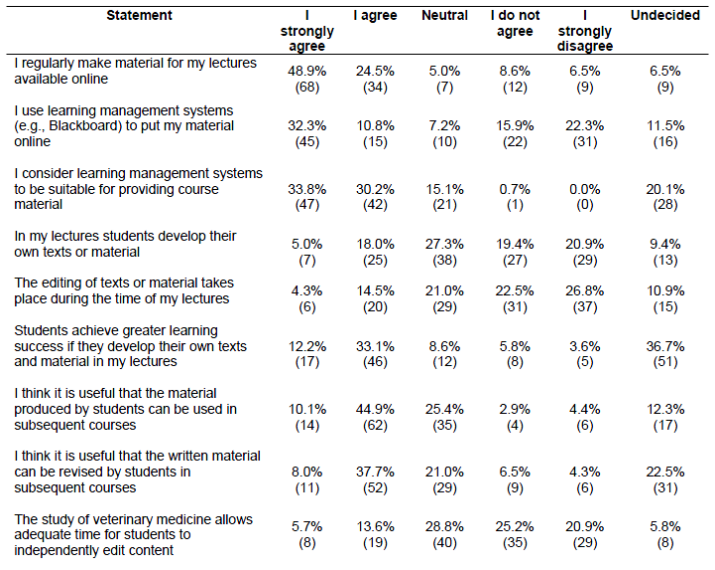
Availability and creation of content in courses

**Table 2:**
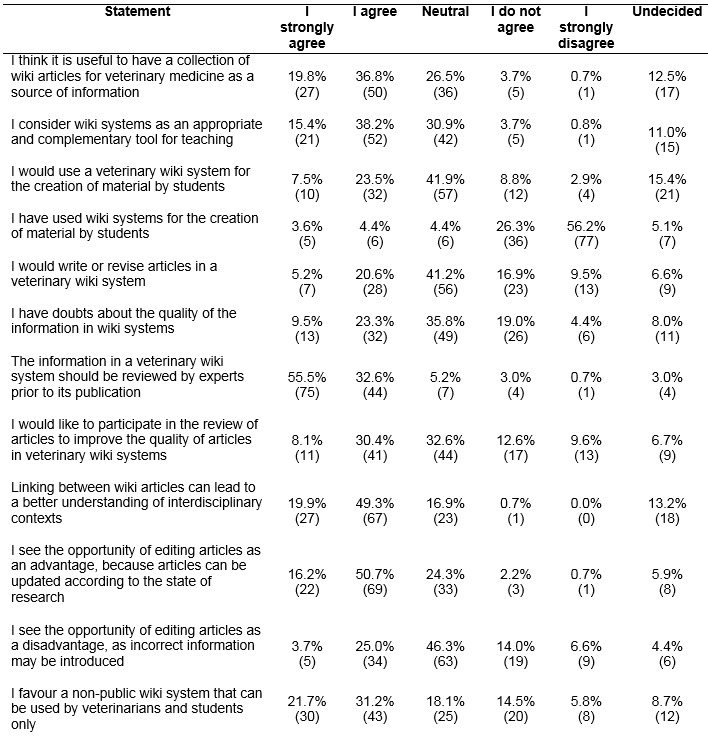
Use and acceptance of wiki systems in courses

**Table 3:**
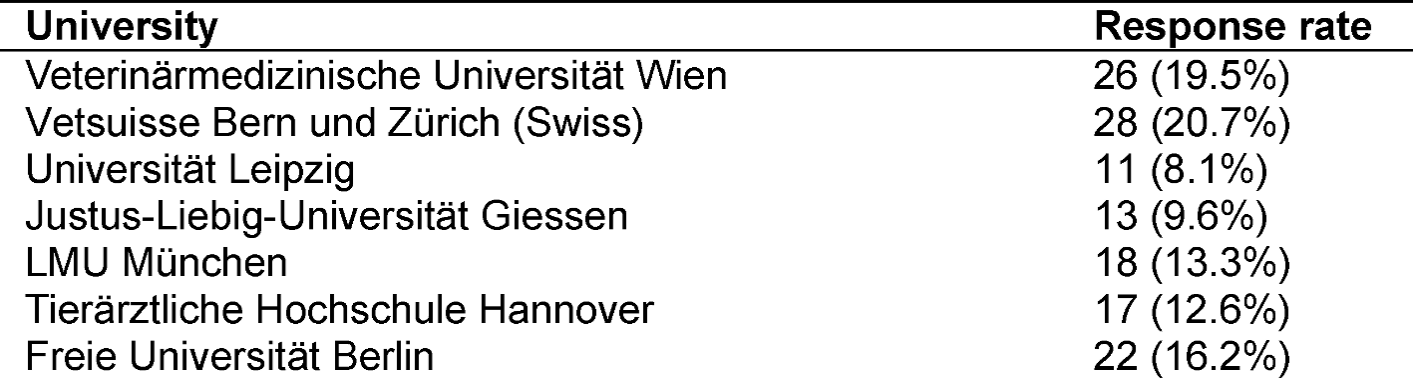
Response rate of the universities
