# Supplementary material for: Use and future of wiki systems in veterinary education? – A survey of lecturers in German-speaking countries
Source: GMS Z Med Ausbild. 2015 Nov 16;32(5):Doc54. doi: 10.3205/zma000996 (PMC4647161; doi:10.3205/zma000996)
Supplement: Questionnaire [file ZMA-32-54-s-001.pdf]

Wiki systems in veterinary education

Dear Lecturers,

in the survey we want to investigate whether you already use wiki systems[1] in veterinary teaching. Furthermore, we are interested in what advantages and disadvantages you see in wiki systems and whether you would play an active role in improving its contents. This survey is being carried out by the veterinary clinic for reproductive medicine at the Freie Universität Berlin (www.tiergyn.de). All information will be treated confidentially and anonymously.

Thank you for your cooperation!

Darius Kolski Dr. Sebastian Arlt Prof. W. Heuwieser

Personal Information

1. Your age? ______
2. You are research assistant ☐ professor ☐
2. What is your area of expertise? ____________________________
3. University: ________________________

1. Questions for lecturers about use and application of wiki systems in the veterinary teaching

Please indicate to what extent you agree or disagree with the statements

**Statement**	I strongly agree	I agree	I moderately agree	I do not agree	I strongly disagree		Undecided
I regularly make material for my lectures available online.	☐	☐	☐	☐	☐		☐
I use learning management systems (e.g. Blackboard) to put my material online	☐	☐	☐	☐	☐		☐
I consider learning management systems to be suitable for providing course material	☐	☐	☐	☐	☐		☐
In my lectures students develop their own texts or material	☐	☐	☐	☐	☐		☐
The editing of texts or material takes place during the time of my lectures	☐	☐	☐	☐	☐		☐
Students achieve greater learning success if they develop their own texts and material in my lectures	☐	☐	☐	☐	☐		☐
I think it is useful that the material produced by students can be used in subsequent courses	☐	☐	☐	☐	☐		☐
I think it is useful that the written material can be revised by students in subsequent courses	☐	☐	☐	☐	☐		☐
The study of veterinary medicine allows adequate time for students to independently edit content	☐	☐	☐	☐	☐		☐

1. **Wiki-System** = software for web sites that contains a collection of linked web pages. These web pages can be developed and edited by individuals or a group of users working collaboratively
2. **Learning-Management-System** = software through which teachers can make course materials etc. available and communicate with students

2. Questions to lecturers about didactic recommendations on the application of wiki systems in university teaching

Please indicate to what extent you agree or disagree with the statements

**Statement**	I strongly agree	I agree	I moderately agree	I do not agree	I strongly disagree		Undecided
I think it is useful to have a collection of wiki articles for veterinary medicine as a source of information	☐	☐	☐	☐	☐		☐
I consider wiki systems as an appropriate and complementary tool for teaching	☐	☐	☐	☐	☐		☐
I would use a veterinary wiki system for the creation of material by students	☐	☐	☐	☐	☐		☐
I have used wiki systems for the creation of material by students	☐	☐	☐	☐	☐		☐
I would write or revise articles in a veterinary wiki system	☐	☐	☐	☐	☐		☐
I have doubts about the quality of the information in wiki systems	☐	☐	☐	☐	☐		☐
The information in a veterinary wiki system should be reviewed by experts prior to its publication	☐	☐	☐	☐	☐		☐
I would like to participate in the review of articles to improve the quality of articles in veterinary wiki systems	☐	☐	☐	☐	☐		☐
Linking between wiki articles can lead to a better understanding of interdisciplinary contexts	☐	☐	☐	☐	☐		☐
I see the opportunity of editing articles as an advantage, because articles can be updated according to the state of research	☐	☐	☐	☐	☐		☐
I see the opportunity of editing articles as a disadvantage, as incorrect information may be introduced	☐	☐	☐	☐	☐		☐
I favor a non-public wiki system that can be used by veterinarians and students only	☐	☐	☐	☐	☐		☐
